# Recognition of *Staphylococcus aureus* by the pattern recognition molecules langerin, mannan-binding lectin, and surfactant protein D: the influence of capsular polysaccharides and wall teichoic acid

**DOI:** 10.3389/fimmu.2024.1504886

**Published:** 2025-01-07

**Authors:** Kirstine Mejlstrup Hymøller, Stig Hill Christiansen, Anders Grønnegaard Schlosser, Uffe B. Skov Sørensen, Jean C. Lee, Steffen Thiel

**Affiliations:** ^1^ Department of Biomedicine, Aarhus University, Aarhus, Denmark; ^2^ The Centre for Cellular Signal Patterns (CellPAT), Aarhus University, Aarhus, Denmark; ^3^ Department of Inflammation Research, Department of Molecular Medicine, University of Southern Denmark, Odense, Denmark; ^4^ Division of Infectious Diseases, Department of Medicine, Brigham and Women’s Hospital and Harvard Medical School, Boston, MA, United States

**Keywords:** innate immunity, langerin, mannan-binding lectin, surfactant protein D, *S. aureus*, C-type lectins, capsular polysaccharides, wall teichoic acid

## Abstract

The innate immune system plays a critical role in the rapid recognition and elimination of pathogens through pattern recognition receptors (PRRs). Among these PRRs are the C-type lectins (CTLs) langerin, mannan-binding lectin (MBL), and surfactant protein D (SP-D), which recognize carbohydrate patterns on pathogens. Each represents proteins from different compartments of the body and employs separate effector mechanisms. We have investigated their interaction with the Gram-positive opportunistic pathogen *Staphylococcus aureus*, a bacterium whose cell wall contains two key glycopolymers: capsular polysaccharide (CP) and wall teichoic acid (WTA). Using a langerin-expressing cell line and recombinant langerin, MBL, and SP-D, we demonstrated that langerin, MBL, and SP-D all recognize nonencapsulated S*. aureus*. However, the bacterium may produce CP that effectively shields *S. aureus* from recognition by all three CTLs. Experiments utilizing mutant *S. aureus* strains confirmed that WTA is a ligand for MBL, but that langerin likely interacts with an additional unknown ligand. A competition assay revealed that MBL and SP-D inhibit langerin’s interaction with *S. aureus*, highlighting the intricate redundancy and cooperation within the innate immune system. This study highlights the dynamic interplay of langerin, MBL, and SP-D in recognizing specific surface structures on *S. aureus* and provides insight into how this pathogen evades innate immune recognition.

## 1 Introduction

The innate immune system constitutes an evolutionarily conserved defense mechanism that reacts immediately to pathogen invasion through cellular and humoral components. A diverse array of pattern recognition receptors (PRRs) detecting conserved molecular patterns on microorganisms is central to this response. An example is the recognition of unusual patterns of surface carbohydrates on microorganisms by carbohydrate-binding proteins of the host. Such recognition enables the immune system to detect pathogens and elicit an appropriate immune response (1). This is crucial for protecting the host from microbial invasion (2, 3).

One superfamily of carbohydrate-binding proteins in mammals is the C-type lectins (CTLs). These are evolutionarily ancient germline-encoded PRRs. They are soluble proteins or membrane-bound receptors, e.g., on the surface of antigen-presenting cells. A characteristic of all CTLs is their carbohydrate recognition domain (CRD), which binds carbohydrates in a calcium-dependent manner (4, 5). The CRD often has either the tripeptide motif EPN (Glu-Pro-Asn) or QPD (Gln-Pro-Asp). The EPN motif leads to a specificity for mannose-type carbohydrates, with recognition of equatorial 3- and 4-hydroxyl groups. The QPD motif confers a galactose-type specificity recognizing a combination of an equatorial 3-OH and an axial 4-OH group (6). The affinity for the individual monosaccharide residues is often very low. High binding strength is achieved by oligomerization of CRDs, increasing the avidity for a fitting pattern of carbohydrates. Hence, oligomerization increases the binding strength and fine-tunes the specificity of the individual CTL (7).

**Figure 1:**
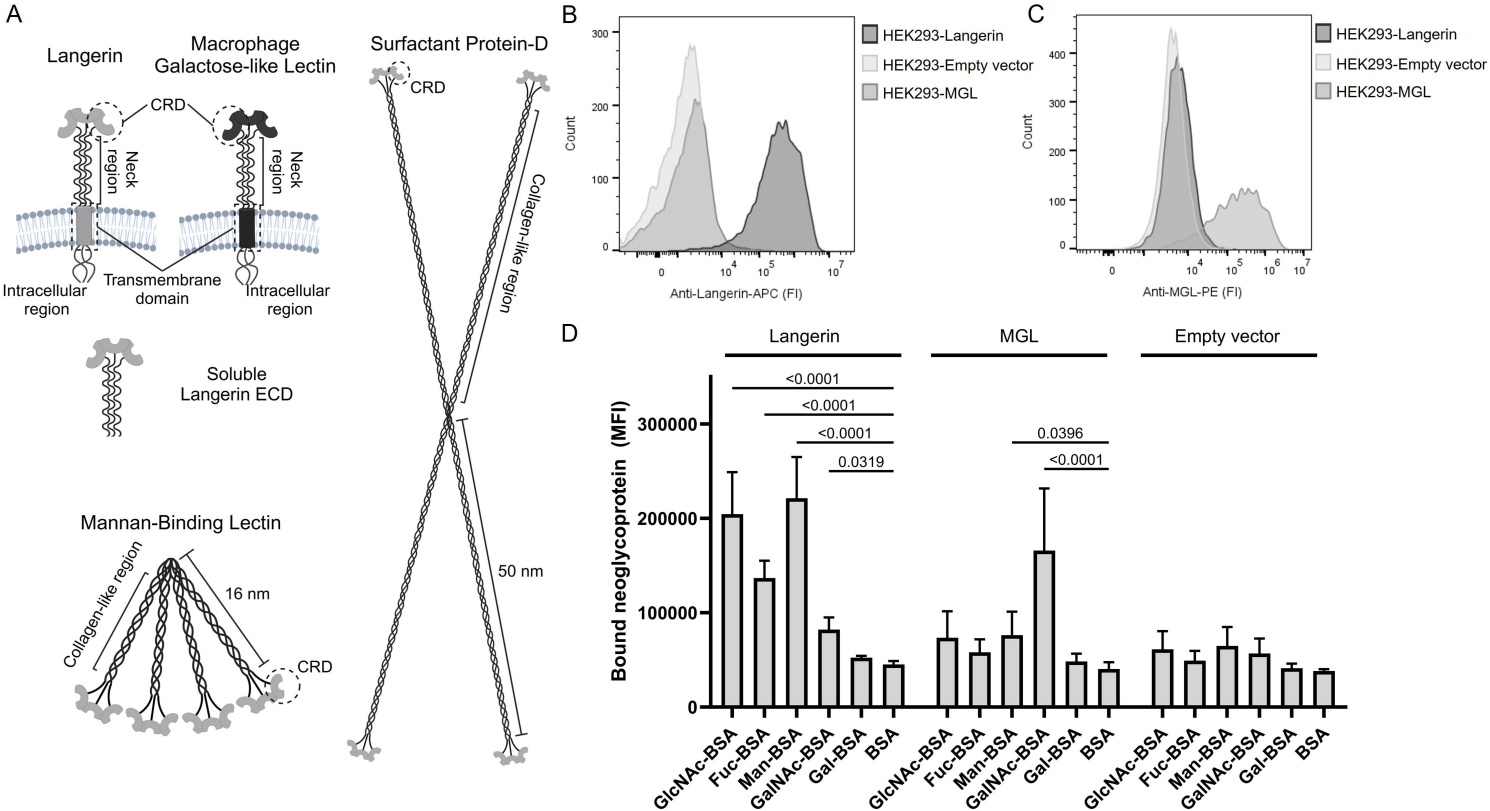
C-type lectins of the innate immune system. **(A)** Illustrations of the five C-type lectins examined in the present study. Langerin and Macrophage galactose-type lectin (MGL) are transmembrane proteins that form alpha-helical coil mediated trimers on the surface of antigen-presenting cells. We produced a recombinant soluble langerin extracellular domain (ECD), which spontaneously forms a trimer. Mannan-binding lectin (MBL) and surfactant protein D (SP-D) form higher-order oligomeric structures through their collagen-like regions. The carbohydrate recognition domains are located at the ends of the structures. The illustrations are not to scale. Created with BioRender.com. **(B)** Validation of langerin expression on HEK293-cells, using APC-labeled specific anti-langerin antibodies. Cell lines with a Langerin encoding vector, MGL encoding vector, or an empty vector are tested. One representative experiment is shown. **(C)** Validation of the expression of MGL on HEK293-cells using PE-labeled specific anti-MGL antibodies. One representative experiment is shown. **(D)** Validation of functional activity and carbohydrate specificity of the Langerin- and MGL-expressing cell lines using various biotinylated neoglycoproteins followed by BV421-labeled streptavidin. Data are presented as the mean of median fluorescence intensity (MFI) +/- sd from seven independent experiments. The data are tested using two-way ANOVA followed by Dunnett’s multiple comparison test. BSA, bovine serum albumin; GlcNAc-BSA, N-acetylglucosamine-BSA; Fuc-BSA, fucose-BSA; Man-BSA, mannose-BSA; GalNAc-BSA, N-acetylgalactosamine; Gal-BSA, galactose-BSA.

We aimed to investigate CTLs of the innate immune system and their interactions with selected microorganisms. In this report, we studied four different CTLs: langerin (also known as CD207 or CLEC4K), macrophage galactose-type lectin (MGL, also known as CD301 or CLEC10A), mannan-binding lectin (MBL, also known as mannose-binding lectin) and surfactant protein D (SP-D). The sizes and conformation of these CTLs are illustrated in **Figure 1A**. These CTLs represent the recognition of diverse patterns of carbohydrates in different compartments of the body: the skin (langerin and MGL), plasma (MBL), and mucosa (SP-D).

Langerin and MGL are both type II transmembrane proteins found on antigen-presenting cells. Langerin is primarily associated with Langerhans cells residing in the epidermis, whereas MGL is expressed by dendritic cells and macrophages, e.g., found in the dermis. Both proteins contain a short intracellular region without classical signaling motifs, a transmembrane region, and an extracellular domain (ECD) consisting of a neck region and a C-terminal CRD (**Figure 1A**). The neck region facilitates trimerization of the proteins through coiled coils of alpha helices (8, 9). Langerin has an EPN-like CRD with, e.g., specificity for mannose, fucose, and N-acetylglucosamine (GlcNAc) (10), whereas MGL has a QPD-based CRD and binds N-acetylgalactosamine (GalNAc) (9). Recognition of a pathogen by these CTLs is proposed to induce endocytosis and killing of the pathogen and presentation to other immune cells for a further immune response (11, 12).

MBL and SP-D are both collectins, i.e., soluble CTLs with a collagen-like domain (**Figure 1A**). MBL is a plasma protein well known for its role in the initiation of the lectin pathway of the complement system (13). As depicted in **Figure 1A**, MBL is a higher order multimer built from trimers of the polypeptide chain. Like Langerin, MBL has a specificity for mannose-like carbohydrates. Binding by MBL can lead to direct complement-mediated killing of pathogens or opsonization and elimination by phagocytosis (13). SP-D has an EPN-based CRD like MBL and langerin. It is primarily expressed in the lungs, but it has also been localized to the gastrointestinal tract and the skin (14). The role of SP-D is aggregation and opsonization of pathogens, but it is also crucial for maintaining alveolar integrity. SP-D can assemble into remarkably large (~100 nm), highly oligomeric structures, such as a cruciform, as shown in **Figure 1A** (15, 16).

These CTLs are thought to interact with a range of different microorganisms, including S*taphylococcus aureus* (2, 3, 17), a Gram-positive commensal bacterium that colonizes the skin, nares, and gastrointestinal tract of humans. It is an opportunistic pathogen and can cause a broad range of infections, including skin and soft tissue infections, pneumonia, and sepsis (18). Thus, the host’s immune system needs to recognize and clear this bacterium when an infection is initiated. The surface layers of *S. aureus* cells contain glycopolymers such as peptidoglycan, lipoteichoic acid (LTA), wall teichoic acid (WTA), capsular polysaccharide (CP), poly N-acetyl glucosamine (PNAG), and glycosylated surface proteins (19–21) (**Supplementary Figure S1**). It has been shown that *S. aureus* WTA is a ligand for both langerin and MBL (22, 23). WTA, a ribitol phosphate glycopolymer modified with D-alanine and GlcNAc, is covalently anchored to *S. aureus* peptidoglycan. In *S. aureus*, this backbone can be modified with GlcNAc residues. Three glycosyltransferases, TarM, TarS, and TarP, are responsible for α-1,4-GlcNAc, β-1,4-GlcNAc, and β-1,3-GlcNAc WTA modifications, respectively (24, 25). It has been reported that langerin can sense the β-GlcNAc but not the α-GlcNAc WTA modification (17, 22), whereas MBL recognizes both modifications (23). The *S. aureus* CP surrounds the bacterium and prevents bacterial recognition by eukaryotic cells, including phagocytes (26–29). The two main capsular serotypes (CP5 and CP8) are both repeating units of D-*N*-acetyl mannosaminuronic acid, L-*N*-acetyl fucosamine, and D-*N*-acetyl fucosamine with distinct linkages between the carbohydrates and the sites of O-acetylation (30).

The present report demonstrates that langerin, MBL, and SP-D can bind to *S. aureus*, but MGL cannot. However, the presence of surface-associated CP can effectively shield *S. aureus* against the interaction with langerin, MBL, and SP-D. Experiments performed with WTA mutant strains confirm that WTA decorated with α-1,4-GlcNAc and/or β-1,4-GlcNAc, serve as ligands for MBL. Our data suggest the presence of an additional unknown ligand for langerin, demanding further investigation. Finally, using a competition assay, we observed that langerin, MBL, and SP-D may compete for binding to *S. aureus*, underscoring the complex network characteristic of the innate immune system in the defense against this bacterium. Our study highlights the crucial roles and interplay of langerin, MBL, and SP-D in recognizing specific surface structures on *S. aureus* and how this bacterium evades recognition.

## 2 Materials and methods

### 2.1 Bacterial strains

The bacterial strains used in the study and their origin are listed in **Table 1**.

**Table 1:** Bacterial strains used in this study.

*Staphylococcus aureus* strains	Reference/source
Reynolds (CP) (GFP)	Watts et al. (31) and Kuipers et al. (26)
Reynolds (CP5) (GFP)	Watts et al. (31) and Kuipers et al. (26)
Reynolds (CP8) (GFP)	Watts et al. (31) and Kuipers et al. (26)
JE2 WT	Fey et al. (32)
JE2 *ΔtagO*	Wang et al. (33)
JE2 *ΔtarM* (NE0611)	BEI resources (NIH/NIAID)
JE2 *ΔtarS* (NE0942)	BEI resources (NIH/NIAID)
JE2 *ΔtarMS*	This study

CP, Capsular polysaccharides; GFP, Green fluorescensens protein; WT Wild type.

### 2.2 Mannan-binding lectin and surfactant protein-D

Preparation of full-length SP-D was performed as described (34). In brief, SP-D was purified by affinity chromatography from the culture supernatant of EXPICHO-S cells transfected with a plasmid encoding SP-D. The production of therapeutic-grade recombinant human MBL has been described previously (35). The recombinant proteins were subjected to reduced or native SDS-PAGE (**Supplementary Figure S2E**) and stained with Coomassie blue.

### 2.3 Expression and purification of recombinant soluble langerin extracellular domain

Recombinant human soluble Langerin ECD (residues 65-328 in Uniprot Q9UJ71) was expressed from the pET30a expression vector carrying kanamycin (KAN) resistance (GenScript, NJ). The vector was transformed into the *Escherichia coli* strain BL21 (DE3) (cat. no. EC0114, Thermo Fisher Scientific). Expression and purification were performed as described (36) with minor modifications. An overnight culture of transformed BL21 was diluted 40 times in Luria-Bertani media with 50 µg/mL kanamycin. The culture was grown at 37°C with agitation to an OD_550_ ≈ 0.5, followed by 1 mM isopropyl-beta-D-thiogalactoside (IPTG) induction. After 3 h, the bacteria were harvested by centrifugation at 4,000 x g for 15 min at 4°C. The pellet was lysed by sonication (10 bursts of 30-sec durations, 45-sec rest) in 10 mM Tris-HCl, pH 7.5. Inclusion bodies were isolated by centrifugation at 10,000 x g for 15 min at 4°C and solubilized by sonication in 6 M guanidine-HCl and 0.01% beta-mercaptoethanol in 100 mM Tris-HCl, pH 7.5, followed by incubation on ice for 30 min. The mixture was centrifuged at 10,000 x g for 30 min at 4°C, and the supernatant was slowly diluted threefold in loading buffer (25 mM Tris-HCl, pH 7.8, 0.15 M NaCl, 25 mM CaCl_2_) to allow protein refolding. The diluted mixture was dialyzed twice against 2 L loading buffer. The insoluble precipitate was removed by centrifugation at 10,000 x g for 30 min at 4°C.

The supernatant containing refolded recombinant human Langerin ECD was loaded onto Toyopearl HW 75f (TSK beads) as a pre-clearing step and incubated at 4°C for 1 h. The mixture was centrifuged for 5 min at 200 x g, and the supernatant was loaded onto mannose-coupled TSK beads, prepared as given in (37) and incubated for 2 h end over end. The beads were washed with loading buffer, and protein was eluted in EDTA-elution buffer (25 mM Tris-HCl, pH 7.8, 150 mM NaCl, 2 mM EDTA). The eluted fractions were analyzed by SDS-PAGE, and the protein concentration was determined through absorbance at 280 nm using a calculated molar extinction coefficient of 56,170 M^-1^ cm^-1^. Fractions containing the expected size of human Langerin ECD (~29 kDa) were pooled and further purified via size exclusion chromatography using a Superdex200 Increase 10/300 column (Cytiva) equilibrated in EDTA-elution buffer. Fractions in the peak signal were analyzed by SDS-PAGE, and fractions D7-E1 were pooled, as shown in **Supplementary Figures S2A, B**.

### 2.4 Labeling with biotin

Neoglycoproteins were obtained from Dextra: fucose-BSA (Fuc-BSA) (cat. no. NGP1105), mannose-BSA (Man-BSA) (cat. no. NGP1108), N-acetyl glucosamine-BSA (GlcNAc-BSA) (cat. no. NGP1101), N-acetyl galactosamine-BSA (GalNAc-BSA) (cat. no. NGP1104), galactose-BSA (Gal-BSA) (cat. no. NGP1107) or BSA (cat. no. 9048-46-8, Sigma-Aldrich). These neoglycoproteins, soluble Langerin ECD, and MBL were dialyzed twice against phosphate-buffered saline (PBS), pH 7.4, and then once against PBS, pH 8.5. Biotin N-hydroxysuccinimide ester (cat. no. H1759, Sigma-Aldrich) was added (167 µg per mg protein) and reacted for 4 h at room temperature before termination of the reaction by three rounds of dialysis against Tris-buffered saline (TBS; 10 mM Tris, 140 mM NaCl, 0.09% (w/v) NaN_3_, pH 7.4).

### 2.5 Generation of C-type lectin expressing cell lines

The Freestyle 293-F cell line (cat. no. R79007, Thermo Fisher Scientific), here referred to as HEK293, was cultured in 6-well plates to 70-90% confluency at transfection in Freestyle 293 Expression Medium (cat. no. 12338001, Thermo Fisher Scientific) with 10% fetal bovine serum (FBS) (cat. no. F7524, Sigma-Aldrich), 100 units/ml penicillin, and 100 µg/ml streptomycin (cat. no. 15140-122, Thermo Fisher Scientific). The HEK293 cell line was replenished with fresh expression medium with 10% FBS on the day of transfection. Expression constructs containing full-length open reading frames for Langerin (accession number NM_015717.5) and MGL (accession number NM_182906.4) were cloned into the mammalian expression vector pcDNA3.1/Zeo+ (GenScript), which confers resistance to Zeocin.

The Langerin- and MGL-encoding vectors and an empty vector (mock, i.e., the pcDNA3.1/Zeo+ vector alone) were introduced into the adherent HEK293 cell line (the cell line is adherent under the conditions used with FBS) by transfection. Briefly, DNA and Lipofectamine 3000 (cat. no. L3000001, Thermo Fisher Scientific) were mixed in two steps following the instruction manual. First, the lipofectamine 3000 reagent was diluted in OptiPRO serum-free medium (SFM) (cat. no. 12309050, Thermo Fisher Scientific). Second, 5 µg plasmid DNA was diluted in OptiPRO SFM, and then 10 µl P3000 reagent was added. The two mixtures were combined and incubated for 10 to 15 min, and DNA-lipid complexes were added to the cells by gently swirling the 6-well plate. The cells were replenished in a fresh culture medium after 24 h of incubation at 37°C and 8% CO_2_. Forty-eight hours after transfection, the cells were supplemented with 500 µg/mL zeocin (cat. no. ant-zn-1, InvivoGen). The culture medium was changed every three days and analyzed for clonal outgrowth after 10-14 days of culture. Resistant clones were separated by limiting dilution into 96-well microtiter plates. Individual clones were screened for lectin expression by flow cytometry (see Confirmation of Langerin or MGL expression), and high expression clones were expanded for cryopreservation in freeze medium (293f Expression Medium with 20% FBS, 10% DMSO).

### 2.6 Cell culture conditions

The HEK293-Langerin, HEK293-MGL, and HEK293-empty vector cells were thawed by resuspension in 293f Expression Medium with 10% FBS. Cells were sedimented by centrifugation at 200 x g and replenished in culture medium. Cells were incubated in standard T75 culture flasks (cat. no. 83.3911.002, Sarstedt) at 37°C and 8% CO_2_. The next day, the culture medium was discarded and replaced with culture medium containing 500 µg/ml zeocin. The cells were split upon 80-90% confluency. Cells were harvested by incubating with PBS with 5 mM EDTA and immediately washed in HBS+ (20 mM HEPES, 150 mM NaCl with 5 mM CaCl_2_, 5 mM MgCl_2_, 0.5% BSA, at pH 7.4) by centrifugation at 200 x g for 5 min. Cell viability and numbers were assessed in the presence of trypan blue (cat. no T8154, Sigma Aldrich).

### 2.7 Confirmation of langerin or MGL expression

The expression of Langerin and MGL was confirmed by antibody staining. HEK293-Langerin, HEK293-MGL, or HEK293-empty vector cell lines (200,000 cells) were incubated with either 4 µg/mL anti-human CD207-APC (clone 10E2, BioLegend) or 6 µg/mL anti-human CD301-PE (clone H037G3, BioLegend) in HBS+. Subsequently, the cells were washed twice before flow cytometry on a NovoCyte 3000 flow cytometer equipped with three lasers (405 nm, 488 nm, and 640 nm) and 13 fluorescence detectors (Agilent, CA). All cells were gated using forward and side scatter height, whereas single cells were gated using forward scatter area and forward scatter height; see the gating strategy in **Supplementary Figure S3A**. Data were analyzed using FlowJO 10 (FlowJo, LLC).

### 2.8 Neoglycoprotein binding assay

The ability of the HEK293-Langerin, HEK293-MGL, or HEK293-empty vector cell lines to bind neoglycoproteins was examined using neoglycoproteins having ~20 monosaccharides coupled per BSA molecule (according to the manufacturer). The cell lines (200,000 cells) were incubated with 10 µg/mL biotin-conjugated neoglycoproteins (Fuc-BSA, Man-BSA, GlcNAc-BSA, GalNAc-BSA, Gal-BSA or BSA) in HBS+ for 30 min at room temperature. The cells were collected by centrifugation at 200 x g and washed twice before incubation with 0.5 µg/mL streptavidin-BV241 (cat. no. 563259, BD Bioscience) in HBS+ for 30 min at room temperature. The cells were subsequently collected by centrifugation and washed twice before flow cytometry was performed, as described above.

### 2.9 Capsule staining of *S. aureus* Reynolds

Green fluorescence protein (GFP) expressing *S. aureus* Reynolds (CP5), Reynolds (CP8), or Reynolds (CP-) were grown overnight at 37°C on Columbia agar with 2% (w/v) NaCl (CSA) or in suspension in Todd Hewitt broth (THB) at 37°C with shaking. The bacteria were harvested by centrifugation in the THB cultures or single colonies picked from the CSA plates. To inhibit non-specific binding, the bacteria were incubated in 1% heat-inactivated goat serum in HBS+ for 45 min at 4°C. Subsequently, the bacteria were incubated for 45 min at 4°C with 1 µg/mL rabbit anti-CP5 (38), 1 µg/mL rabbit anti-CP8 (38), or 10 µg/mL rabbit IgG (control) (cat. no. 7406404, Lampire biological laboratories) in HBS+, 1% goat serum and washed twice with HBS+, 0.2% goat serum. The bacteria were subsequently incubated with 2.5 µg/mL BV421 polyclonal goat anti-rabbit IgG (cat. no. 565014, BD Bioscience) for 45 min at 4°C and washed twice in HBS+, 0.2% goat serum before flow cytometry analysis with the Novocyte 3000 flow cytometer. The GFP-positive events were gated as bacteria; see the gating strategy in **Supplementary Figure S3B**. The data were analyzed using FlowJO 10.

### 2.10 Generation of an *S. aureus* Δ*tarMS* mutant strain

We utilized the Nebraska transposon mutant library (32), comprising derivatives of *S. aureus* JE2, a community-associated methicillin-resistant USA300 LAC strain cured of three plasmids. The library consists of approximately 2,000 mutants, each with one nonessential gene disrupted by insertion of the mariner transposon *bursa aurealis* harboring an erythromycin (Em) resistance gene. The library includes transposon-mutants of *tarS* (NE0942) and *tarM* (NE0611), referred to as JE2Δt*arS* and JE2Δ*tarM*. To generate a JE2Δ*tarMS* mutant, allelic exchange was performed as described in Bose et al. (39) to exchange the Em resistance cassette to Kan in JE2Δ*tarS*. To create the JE2Δ*tarMS* mutant, the Δ*tarM* mutation was transduced to JE2Δ*tarS* using φ80α with selection for Em resistance. Mutants were confirmed by their antibiotic resistance and by PCR using the primers listed in **Table 2**. Bacterial growth rates and hemolysis on sheep blood agar plates were identical for the wild type (WT) and mutant strains.

**Table 2:** Primers used in the study.

Primer	Sequence
Cat FWD	GAACTGGTTACAATAGCGACGG
Cat REV	TCCTGCATGATAACCATCACAA
TarS FWD	GCCGTCAAGTGAGCGTTTAG
TarS REV	GTGGTACACCACGACCATTAAC
TarM FWD	TGGTATGACCTCTTCGATGTTC
TarM REV	TCCCTGGTCCATCACAAATC

### 2.11 Effect of capsular polysaccharide on C-type lectin binding to *S. aureus*

GFP-expressing *S. aureus* Reynolds (CP5), Reynolds (CP8), or Reynolds (CP-) were cultivated overnight at 37°C on CSA or in THB. The bacterial suspensions were adjusted to 0.5 McFarland (McF) in HBS+. The HEK293-Langerin or -the empty vector cells (200,000 cells) were incubated with the GFP-expressing *S. aureus* strains for 30 min at room temperature. The cells were washed twice in HBS, 5 mM MgCl_2_, 5 mM CaCl_2_, and 0.5% BSA, and binding was measured using the NovoCyte 3000 flow cytometer. HEK293 cells were gated as described above, and the percentage of GFP-positive HEK293 cells was determined using FlowJO 10.

To examine the effect of the *S. aureus* CP on the binding of MBL or langerin ECD, GFP-expressing Reynolds (CP5), Reynolds (CP8), and Reynolds (CP-) were cultivated on CSA and adjusted to 0.5 McF in TBS, 5 mM MgCl_2_, 5 mM CaCl_2_, 0.5% BSA (TBS+) or TBS, 10 mM EDTA, 0.5% BSA. The bacteria were incubated with 5 µg/mL biotinylated langerin ECD or 1 µg/mL biotinylated MBL for 30 min, followed by a wash of the bacteria with either TBS+ or TBS, 10 mM EDTA, 0.5% BSA before incubation with streptavidin-BV421 in TBS+. The treated bacteria were subsequently washed twice. The BV421 signal was measured by flow cytometry using the NovoCyte 3000 flow cytometer and analyzed using FlowJO 10, where GFP-positive events were gated as bacteria.

The effect of the *S. aureus* CP on the interaction with SP-D was investigated by assessing bacterial aggregation induced by SP-D [protocol modified from (16)]. The degree of aggregation was measured by measuring light transmission through a bacterial suspension before and after the addition of SP-D. GFP-expressing *S. aureus* Reynolds (CP5), Reynolds (CP8), or Reynolds (CP-) were cultivated on CSA and adjusted to an OD_650_ of 0.4 in TBS + 0.09% NaN_3_. SP-D was prepared in either TBS, 5 mM MgCl_2_, and 5 mM CaCl_2_ or TBS and 10 mM EDTA buffers. Samples prepared in spectrophotometer cuvettes included 400 µL of the bacterial suspension and 100 µL SP-D (to a final concentration of 1 µg/mL) or a control with no SP-D. OD_650_ was measured at the start of incubation and again after 16 h of incubation at 4°C. A decrease in optical density indicates aggregation of bacteria, which will sink in the cuvette and thus disappear from the optical path. The percent aggregation was calculated as $\left(1-\frac{OD_{650\;}16\;h}{OD_{650\;}0\;h}\right)\cdot 100$ .

### 2.12 Effects of wall teichoic acids on lectin binding to *S. aureus*

To examine the influence of wall teichoic acids on lectin binding to *S. aureus*, strains JE2 WT, Δ*tarS*, Δ*tarM*, Δ*tarMS*, and Δ*tagO* were cultivated in THB at 37°C with agitation and stained with FITC. Overnight cultures were subcultured in fresh THB and cultivated to an OD600 ~0.6. The bacteria were washed by centrifugation and resuspended in 100 mM NaHCO_3_ buffer, pH 9, containing 3.33 µg/mL fluorescein isothiocyanate (FITC) (cat no. F1906, Invitrogen). After incubation for 30 min at 37°C in the dark, the bacteria were washed twice in TBS. The mean labeling intensity of the bacteria is shown in **Supplementary Figure S3C** as determined by flow cytometry. The bacteria were resuspended in TBS+ to an OD_600_ of 0.4, equivalent to ~10^8^ CFU/ml. HEK293-Langerin, -MGL or -empty vector cells (200,000 cells) were stained with a 1:2000 dilution of LIVE DEAD™ Fixable Near-IR Dead Cell Stain Kit (cat. no L10119, Invitrogen) for 30 min, washed, and incubated for 30 min at 37°C with the FITC-labelled bacteria at bacteria to cell ratios ranging from 1 to 9. Cells were subsequently washed in TBS+ and fixed in 1% formaldehyde in PBS for 15 min. *S. aureus* binding was measured using a Cytek Northern Lights flow cytometer (Cytek Biosciences, CA), a full spectrum flow cytometer equipped with three lasers (405 nm, 488 nm, and 640 nm). Unmixing of the spectral flow data was performed using a single stain of LIVE DEAD™ Fixable Near-IR Dead Cell Stain Kit of heat-killed cells, FITC-labelled bacteria, and BV421-FC beads (cat. No. 661627, BD Bioscience) using the SpectroFlo software (Cytek Biosciences). Unmixed data were analyzed using FlowJO 10.

Similarly, we examined the influence of the wall teichoic acid structure on the binding of soluble langerin ECD and MBL to fluorescent bacteria. *S. aureus* JE2 WT, Δ*tarS*, Δ*tarM*, Δ*tarMS*, and Δ*tagO* were cultivated and labeled with FITC as described above. Bacteria with an OD_600_ of 0.4 in TBS+ or TBS, 10 mM EDTA, and 0.5% BSA were incubated with decreasing biotin-langerin ECD or biotin-MBL concentrations for 30 min at 37°C. Subsequently, the bacteria were washed twice and incubated with 0.5 µg/mL streptavidin-BV421 (BD Bioscience). The bacteria were washed and fixed in 1% formaldehyde in PBS for 15 min. The BV421 signal was measured using a Cytek Northern Lights flow cytometer. Unmixing of the spectral flow data was performed using FITC-labelled bacteria and BV421-FC beads, as described above.

### 2.13 Competition of MBL and SP-D with langerin

GFP-expressing *S. aureus* Reynolds (CP-) was cultivated overnight on CSA at 37°C. Single colonies were picked and resuspended to 1 McF in HBS+. The bacteria were incubated with increasing amounts of MBL, SP-D, or human serum albumin (HSA) (cat. no. 109697, CSL Behring) for 20 min at room temperature, followed by incubation with either HEK293-Langerin or HEK293-empty vector cell lines for 30 min at room temperature. The cells were washed twice in HBS+. The signal from the GFP-labelled bacteria was detected using the NovoCyte 3000 flow cytometer, as described above.

### 2.14 Statistical analysis

For statistical analysis, normal distribution was assessed using qq-plots. A two-way analysis of variance (ANOVA) followed by Dunnett’s multiple comparison tests was performed in all cases where more than two groups were compared. Except in the SP-D aggregation assay, two-way ANOVA followed by Tukey’s multiple comparison test was used. Unpaired two-sided t-tests analyzed data to compare two groups. Before a t-test, an F-test was used to determine whether the variance was equal between groups. If there was equal variance, the data were analyzed by unpaired two-sided t-test; if not, Welch correction was utilized. Error bars are depicted as mean ± standard deviation (sd) in all bar graphs. Statistical analyses were performed using GraphPad Prism version 10. A p-value below 0.05 was considered significant.

## 3 Results

### 3.1 Generation and characterization of cell-lines expressing C-type lectins

A panel of different soluble and membrane-bound CTLs was generated and characterized (**Figure 1A**). For the membrane-bound CTLs, the HEK293 cell line was employed to generate cell lines constitutively expressing either langerin or MGL; a cell line transfected with an empty vector served as a negative control. The expression of langerin or MGL by the HEK293-cell line was confirmed by flow cytometry using specific antibodies (**Figure 1B, C**). The functional activity and specificity of the two membrane-bound lectins were characterized using BSA conjugated with different monosaccharides, i.e., neoglycoproteins. HEK293-Langerin cells exhibited significantly (P<0.0001) higher binding to GlcNAc-BSA, Man-BSA, and Fuc-BSA than to BSA. For the MGL-expressing HEK293-cells, we observed a highly significant (P<0.0001) specificity for GalNAc-BSA (**Figure 1D**). The specificities we find are consistent with the carbohydrate specificities reported in the literature (9, 10).

### 3.2 Screening of microorganisms for binding by langerin and MGL

The langerin- and MGL-expressing HEK293 cells were used to screen for possible recognition of a range of different strains of *S. aureus* and *Streptococcus pneumoniae*. A collection of laboratory *S. aureus* strains (T1-T13 and Wood) was initially examined. HEK293-Langerin, compared to the empty vector control, showed binding of all strains (**Supplementary Figure S4**). However, we observed a marked variation ranging from less than 20 percent to almost 80 percent of *S. aureus-*positive HEK293-Langerin cells. The MGL-expressing cells, on the other hand, did not bind any of the *S. aureus* strains examined. A collection of 89 different *S. pneumoniae* serotypes (i.e., representing 89 different capsular structures) and two nonencapsulated strains (ATCC12213 and C-mutant) was also screened for binding to HEK293-Langerin and HEK293-MGL. No binding of any of the *S. pneumoniae* strains was observed for either the HEK293-Langerin (despite good binding to the positive control *S. aureus* Wood) or the HEK-293-MGL cells (**Supplementary Figure S5**). These results demonstrate that Langerin, but not MGL, can recognize *S. aureus*; neither Langerin nor MGL recognized any of the *S. pneumoniae* serotypes tested.

### 3.3 The effect of growth conditions on capsular polysaccharide expression and langerin recognition

Others have reported the interaction between langerin and *S. aureus* (22). However, the majority of *S. aureus* strains produce CP, which is highly dependent on the bacterial culture conditions (40). CP is a staphylococcal virulence factor that is antiphagocytic and has been reported to mask surface antigens, evading recognition by PPRs of the innate immune system (26–29). Thus, we wanted to examine the influence of growth conditions and production of CP on the recognition of *S. aureus* by langerin.

**Figure 2:**
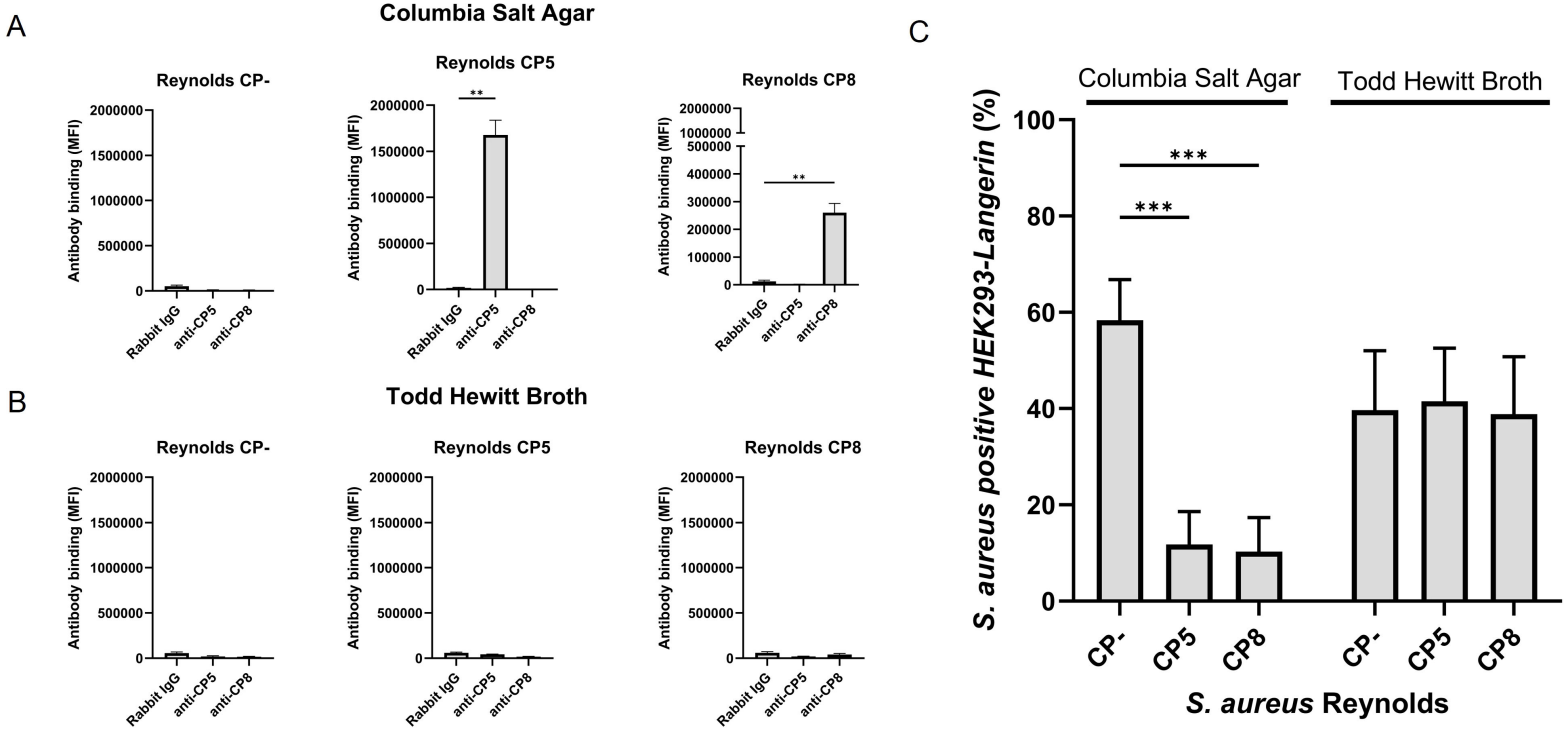
The influence of capsular polysaccharide expression on the recognition of *S. aureus* by langerin. *S. aureus* Reynolds (CP-), Reynolds (CP5), or Reynolds (CP8), expressing green fluorescent protein (GFP), were cultivated **(A)** on Columbia salt agar (CSA) or **(B)** in Todd Hewitt broth (THB). CP expression was detected by flow cytometry with either rabbit anti-CP5, rabbit anti-CP8 antibodies, or normal rabbit IgG (negative control), followed by detection with labeled goat anti-rabbit IgG. Antibody binding is presented as the mean of MFI +/- sd of three independent experiments, **p < 0.01, two-sided t-test. **(C)** GFP-expressing *S. aureus* Reynolds (CP-), Reynolds (CP5), or Reynolds (CP8) were cultivated on CSA or in THB and tested for binding to HEK293-Langerin cells. Data are presented as percent Langerin-expressing cells positive for *S. aureus* (GFP) with HEK293-empty vectors subtracted, mean +/- sd of three independent experiments, ***p < 0.001, two-way ANOVA followed by Dunnett’s multiple comparison test.

*S. aureus* CP serotypes 5 and 8 represent at least 80% of clinical isolates (41). *S. aureus* Reynolds is a well-characterized strain for assessing the biological properties of CP; it produces CP5. To study the effect of CP on langerin binding, two isogenic mutants, a Reynolds strain producing CP8 and a strain with no CP production (CP-), were additionally employed (31). The degree of CP production was examined by the reaction of specific CP5 and CP8 antibodies with *S. aureus* strains cultivated either on CSA or in THB. On CSA, both Reynolds (CP5) and Reynolds (CP8) produced abundant CP5 and CP8, respectively, while Reynolds (CP-) did not produce CP (**Figure 2A**). When cultivated in THB, none of the three serotypes produced detectable amounts of CP (**Figure 2B**). We thus confirmed the observation that little CP is associated with staphylococcal cells in broth cultures (29, 42).

To study whether this difference in CP production influenced the interaction between langerin and *S. aureus*, GFP expressing Reynolds (CP5), Reynolds (CP8), and Reynolds (CP-) were cultivated on CSA or in THB. The bacteria were incubated with HEK293-Langerin, and the percentage of *S. aureus-*positive HEK293 cells was measured. When cultivated on CSA, both Reynolds (CP5) and Reynolds (CP8) showed a significant decrease in the interaction with HEK293-Langerin compared to Reynolds (CP-), from 58% *S. aureus* positive HEK393-Langerin cells to 11% for Reynolds (CP5) and 10% for Reynolds (CP8) (**Figure 2C**). Conversely, all three serotypes exhibited equal interaction with HEK293-Langerin when cultivated in THB. The results suggest that the CP effectively masks the surface of *S. aureus* cells for recognition by this innate immune system recognition molecule.

### 3.4 Capsular polysaccharide production and recognition by soluble C-type lectins

**Figure 3:**
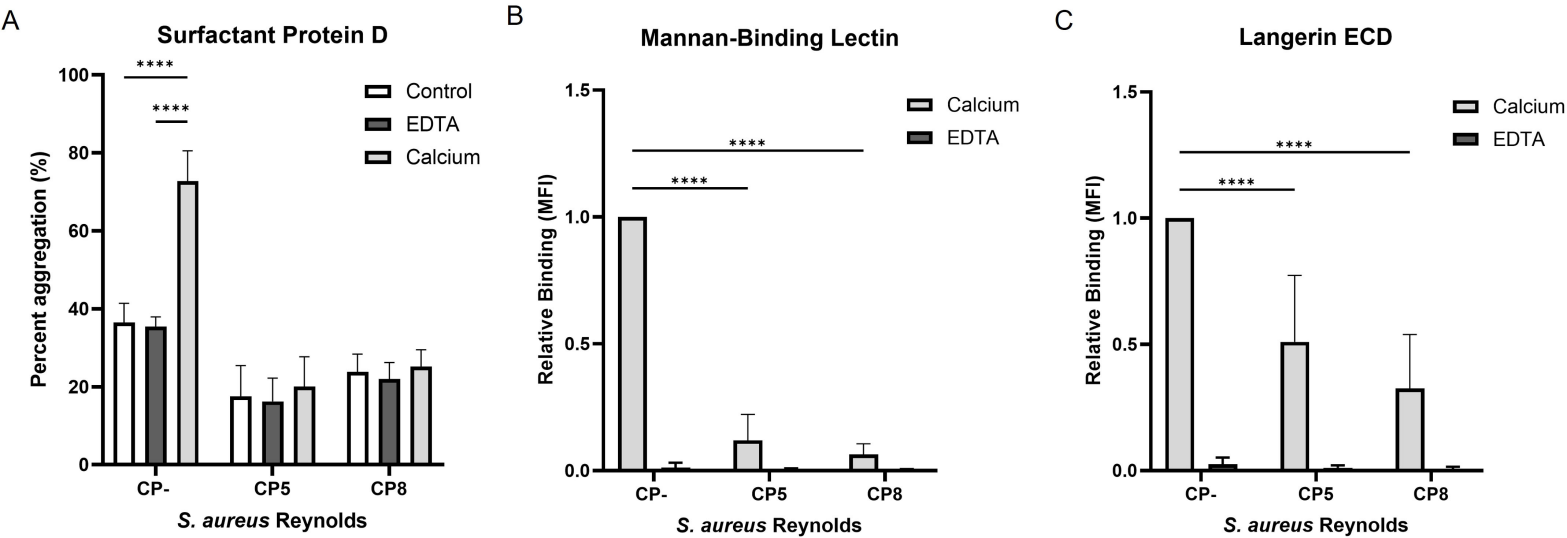
The influence of capsular polysaccharides expression on the recognition of *S. aureus* by Surfactant protein D, mannan-binding lectin, and langerin ECD. GFP-expressing *S. aureus* Reynolds (CP-), Reynolds (CP5), or Reynolds (CP8) were cultivated on CSA. **(A)** Bacterial aggregation by SP-D. Bacteria incubated with or without SP-D (control) in either calcium or EDTA. Percent aggregation was determined as the difference between the initial absorbance measured at OD650 and after 16 h. Data are presented as the mean percent aggregation +/- sd of three independent experiments, ****p < 0.0001, two-way ANOVA followed by Tukey’s multiple comparison test. **(B)** Binding of biotinylated mannan-binding lectin and **(C)** Binding of biotinylated Langerin ECD to the bacteria in either calcium or EDTA. The recognition of carbohydrates by CTLs is calcium dependent. Data are presented normalized to CP- and depicted as the mean +/- sd, Langerin; five independent experiments, Mannan-binding lectin; four independent experiments, ****p < 0.0001, two-way ANOVA followed by Dunnett’s multiple comparison test.

Hereafter, we assessed the influence of CP on the interaction of SP-D and MBL with *S. aureus*. Since the role of SP-D in the defense against pathogens is bacterial aggregation, we investigated the ability of SP-D to aggregate *S. aureus*. Bacterial suspensions of either Reynolds (CP5), Reynolds (CP8), or Reynolds (CP-) cultivated on CSA were incubated with SP-D in the presence of either calcium or EDTA, since the binding of SP-D is calcium dependent. The degree of aggregation was determined by measuring the light absorbance of the sample before and after 16 hours of incubation. As shown in **Figure 3A**, SP-D aggregated Reynolds (CP-) in a calcium-dependent manner (73% aggregation vs. 35% aggregation in the presence or absence of calcium, respectively). The encapsulated strains Reynolds (CP5) and Reynolds (CP8) failed to aggregate in the presence of SP-D.

The influence of CP on the interaction of MBL with *S. aureus* was investigated using flow cytometry. Reynolds (CP5), Reynolds (CP8), and Reynolds (CP-) cultivated on CSA were incubated with biotinylated MBL in the presence of either calcium or EDTA. MBL binding was detected by flow cytometry using streptavidin-BV421 to detect bound biotinylated MBL. As seen for bacterial aggregation by SP-D, MBL bound Reynolds (CP-) in a calcium-dependent manner, whereas MBL binding to both Reynolds (CP5) and Reynolds (CP8) was significantly decreased (**Figure 3B**). Although both SP-D and MBL interact with *S. aureus* in a calcium-dependent manner, bacteria that produce CP avoid aggregation by SP-D and recognition by MBL.

Finally, we examined the binding of a soluble form of langerin to *S. aureus.* Recombinant soluble langerin extracellular domain (langerin ECD) was expressed in *E. coli* and purified using mannose-coupled beads. This was followed by fractionation by size-exclusion chromatography (SEC). Through this procedure, we obtained a high-purity preparation of langerin ECD that eluted from the SEC column as a homogenous peak at ~90 kDa (**Supplementary Figures S2A, B**). This protein langerin ECD formed an expected trimer of ~90 kDa, as detected by mass photometry (**Supplementary Figure S2C**). Furthermore, the functionality of langerin ECD was examined, and as shown in **Supplementary Figure S2D**, langerin ECD bound mannose-BSA but not GalNAc-BSA or BSA. The langerin ECD was incubated in the presence of calcium or EDTA with Reynolds (CP5), Reynolds (CP8), or Reynolds (CP-) cultivated on CSA. We observed that langerin ECD binding was impaired by the presence of CP, i.e., a significant decrease in binding was observed for the encapsulated strains compared to Reynolds (CP-) (**Figure 3C**). Moreover, the addition of EDTA completely abolished langerin ECD binding (**Figure 3C**). The binding of langerin was blocked by the capsule, both when langerin was tested as a membrane-bound (**Figure 2C**) or as a soluble molecule (**Figure 3C**).

### 3.5 Wall teichoic acid as a ligand for mannan-binding lectin and langerin

**Figure 4:**
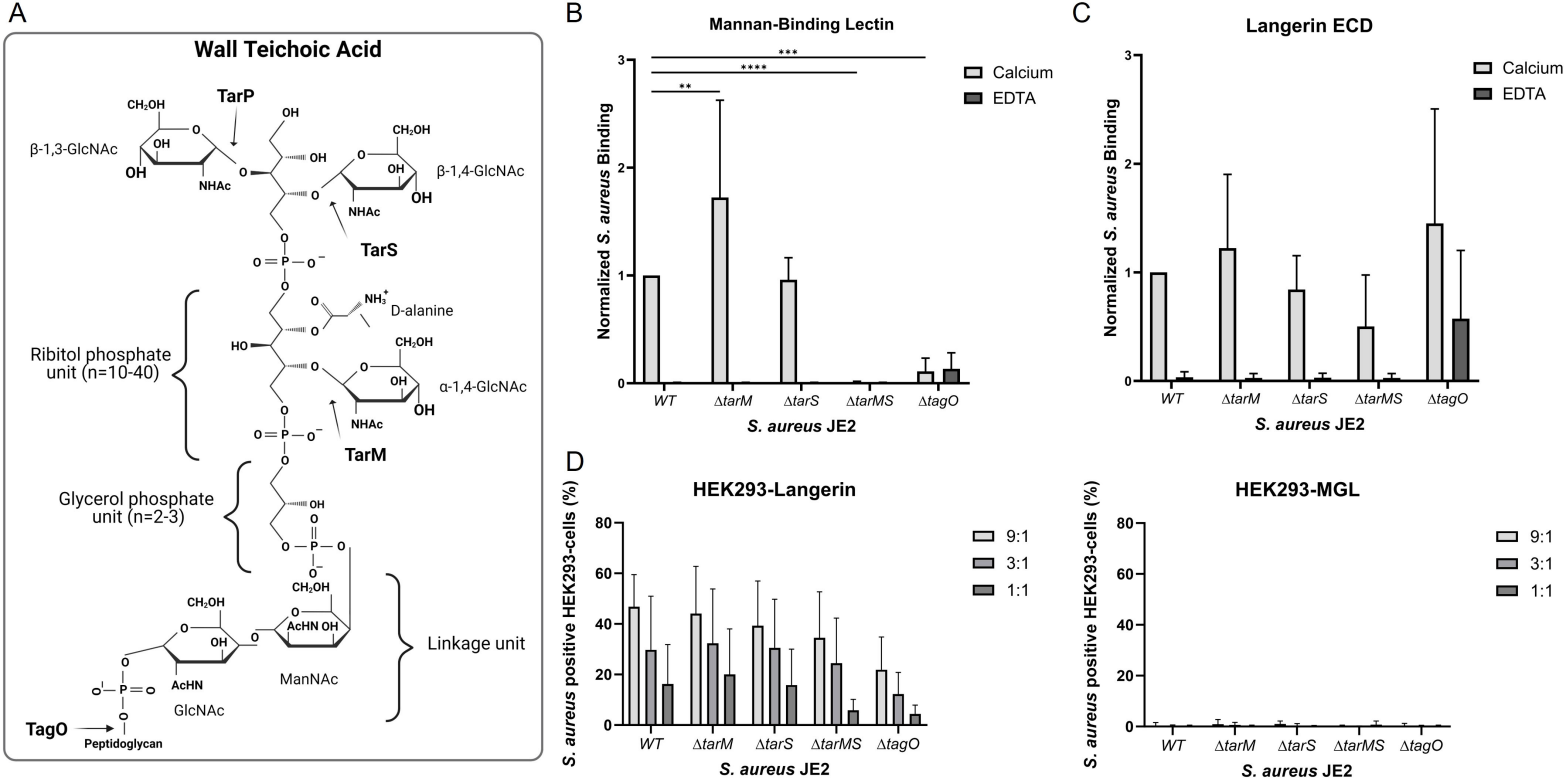
The influence of various wall teichoic acid (WTA) structures on the Langerin and MBL recognition of *S. aureus*. **(A)** WTA is comprised of repeating units of ribitol phosphate modified by D-alanine and either α-1,4-GlcNAc, β-1,4-GlcNAc, or β-1,3-GlcNAc, which are added to the backbone by the glycosyltransferases TarM, TarS or TarP, respectively. The WTA is anchored to the peptidoglycan layer through a covalent linkage unit with glycerol phosphate and a ManNac-GlcNAc unit. The synthesis of WTA is initiated by the enzyme TagO. **(B)** Binding of biotinylated mannan-binding lectin (0.5 µg/ml) and **(C)** biotinylated langerin ECD (4.5 µg/mL) to *S. aureus* JE2 WT or WTA mutants *ΔtarM, ΔtarS, ΔtarMS*, and *ΔtagO*. The binding assay was performed in the presence of either calcium or EDTA. The data is normalized to JE2 WT and depicted as mean +/- sd of calcium; six independent, EDTA; three independent experiments, **p < 0.01, ***p < 0.001, ****p<0.0001, two-way ANOVA followed by Dunnett’s multiple comparison test. **(D)** Binding of HEK293 cells expressing either langerin or MGL to FITC-labelled *S. aureus* JE2 WT or WTA mutants *ΔtarM, ΔtarS, ΔtarMS*, or *ΔtagO.* 9:1, 3:1, and 1:1 indicate the bacteria-to-cell ratio. The data are presented as percent cells positive for FITC with background binding to HEK293-empty vector subtracted, mean of MFI ± sd, of four independent experiments, two-way ANOVA followed by Dunnett’s multiple comparison test.

WTA has been shown to be a target on *S. aureus* for langerin and MBL (22, 23). WTA is an important constituent of the cell wall of Gram-positive bacteria. *S. aureus* WTA is a glycopolymer of ribitol phosphate repeating units modified with D-alanine and anchored to peptidoglycan (**Figure 4A**). Three glycosyltransferases, TarM, TarS, and TarP, are responsible for α-1,4-GlcNAc, β-1,4-GlcNAc, and β-1,3-GlcNAc modifications, respectively (**Figure 4A**). TarS is the most common glycosyltransferase in *S. aureus*. In some cases, TarS is co-expressed with TarM and, in rare cases, with TarP (25). It has been shown that langerin senses the β-GlcNAc but not the α-GlcNAc modification on WTA (17, 22), whereas MBL recognizes both modifications (23).

We utilized the *S. aureus* Nebraska transposon library (32) constructed in strain JE2, a capsule-negative isolate (43) in our experiments. JE2 NE0942 carries an insertion in *tarS*, and JE2 NE0611 carries an insertion in *tarM*, herein called JE2*ΔtarS* and JE2*ΔtarM*. To further examine the dependence of GlcNAc for langerin and MBL binding to *S. aureus*, a double mutant JE2*ΔtarMS* lacking both the TarS and TarM glycosyltransferases, was constructed. This strain lacks GlcNAc modifications of WTA since JE2 only expresses *tarS* and *tarM*. The JE2*ΔtagO* mutant lacks WTA since TagO is the enzyme responsible for initiating WTA biosynthesis (44).

The binding of MBL and langerin ECD to *S. aureus* JE2 WT and its WTA mutants in the presence of either calcium or EDTA was analyzed by flow cytometry. MBL binding was significantly increased for the JE2*ΔtarM* mutant compared to the WT strain, whereas there was an apparent loss of binding to the JE2*ΔtarMS* and JE2*ΔtagO* mutants (**Figure 4B**). This finding was confirmed when a range of different MBL concentrations in the binding reaction was tested (**Supplementary Figure S6A**). In contrast, langerin ECD showed only modest differences in binding to the WT strain JE2 and its WTA mutants (**Figure 4C**). Similarly, few differences were observed when different concentrations of langerin ECD were tested in the binding reaction (**Supplementary Figure S6B**). The JE2 *ΔtagO* mutant bound the langerin ECD in the presence or absence of calcium. These results are not consistent with previously published results (17, 22) since we observed only an indication of WTA-dependent langerin binding and suggest that langerin binds to an additional *S. aureus* ligand.

Previous reports measured the interaction between soluble recombinant langerin and *S. aureus* (17, 22). To measure the interactions between langerin-expressing cells and the different WTA mutants, HEK293-Langerin, HEK293-MGL, or HEK293-empty vector cells were incubated with FITC-labeled JE2 or its WTA mutants. Langerin binding to the HEK293 cells was expressed as the percentage of HEK293 cells that stained positive for *S. aureus* as detected by flow cytometry. Whereas embrane-bound langerin interacted with JE2 WT, JE2 *ΔtarS*, JE2 *ΔtarM*, JE2 *ΔtarMS*, and JE2 *ΔtagO* in a concentration-dependent manner (**Figure 4D**). No significant difference between the WT and the different mutants was observed. Consistent with our Langerin ECD experiments, our studies with HEK293-Langerin cells indicate an additional ligand for Langerin on *S. aureus* besides the β-1,4-GlcNAc modifications on WTA, as suggested by others (17, 22). HEK293-MGL did not exhibit binding to any of the *S. aureus* strains (**Figure 4D**).

### 3.6 Different C-type lectins of the innate immune system can compete for binding to *S. aureus*

**Figure 5:**
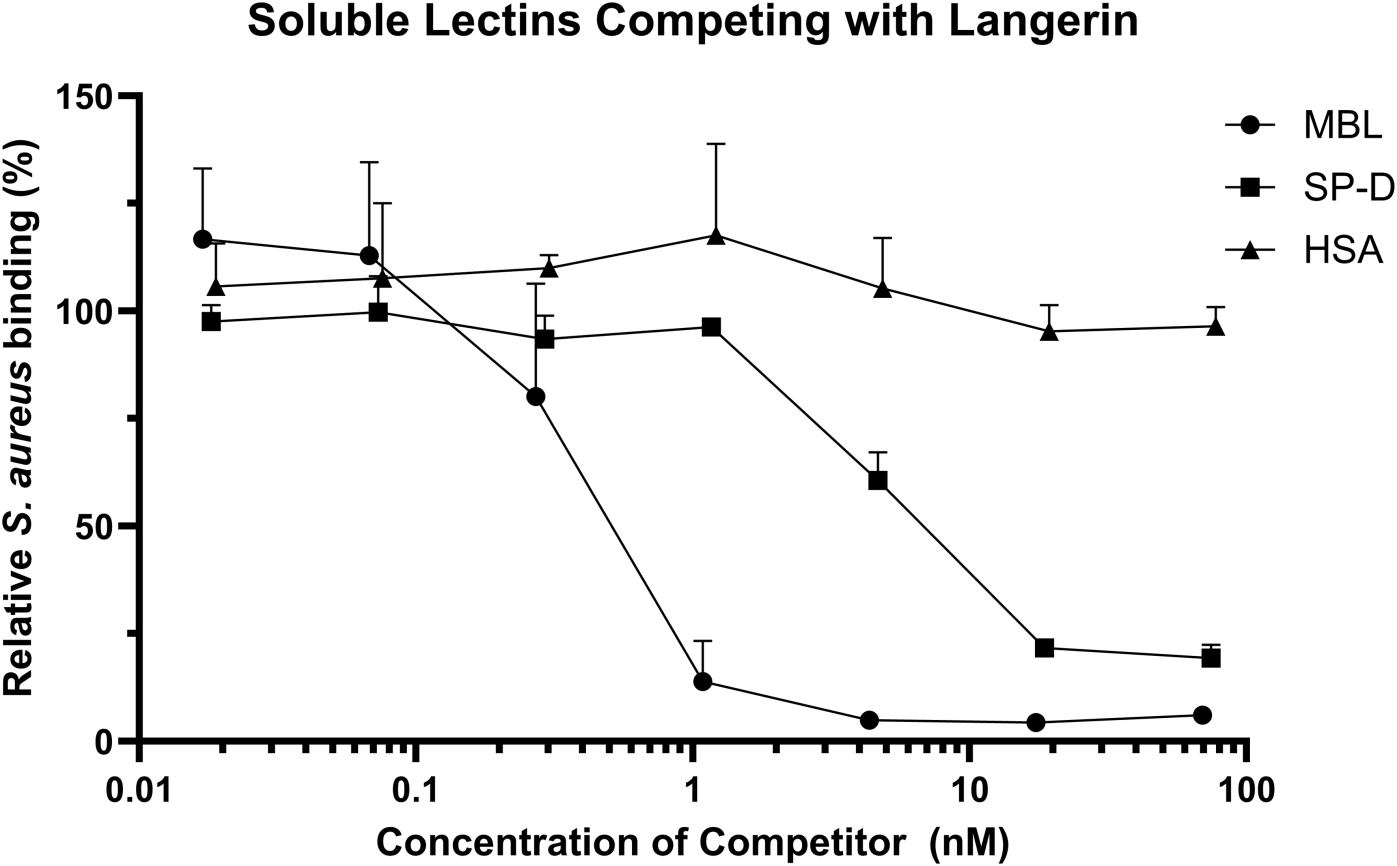
Soluble C-type lectins compete with Langerin. GFP-expressing *S. aureus* Reynolds (CP-) was incubated with either MBL, SP-D, or human serum albumin (HSA) before incubation with HEK293-Langerin cells, followed by detection of *S. aureus* binding by flow cytometry. The data is presented as *S. aureus* binding with inhibitor present relative to HEK293-Langerin binding without addition of inhibitor. Each symbol represents the mean + sd of MBL four, SP-D three, and HSA, two independent experiments, respectively. The symbol covers some error bars.

Langerin, MBL, and SP-D all have comparable carbohydrate specificities, and our data indicate that they each interact with *S. aureus.* We addressed whether these similar CTLs compete for binding to *S. aureus*. GFP-expressing Reynolds (CP-) bacteria were incubated with HEK293-Langerin or HEK293-empty control with the addition of increasing concentrations of either MBL, SP-D, or human serum albumin (HSA). The percentage of *S. aureus*-positive cells was determined by flow cytometry. As shown in **Figure 5**, both MBL and SP-D had inhibitory effects on the interaction between langerin and *S. aureus*, whereas HSA did not influence langerin binding. The data were fitted using four-parameter logistic regression to determine an IC50 for the two soluble CTLs. The IC50 for MBL was 0.407 nM, 95% CI [0.257, 0.557] and R^2^ = 0.9210, whereas the IC50 of SP-D was 4.86 nM, 95% IC [4.164, 5.556] and R^2^ = 0.9732. Thus, MBL was almost ten times better in the competition assay than SP-D. This might be due to a stronger interaction between MBL and *S. aureus* than SP-D and *S. aureus*. In conclusion, these data show that different lectins with similar affinities can compete for the same ligands in a complex natural setting.

## 4 Discussion

A quick and efficient immune response requires the recognition of bacterial pathogens through PRRs of the innate immune system. Langerin, MBL, and SP-D have all been suggested to be involved in the initial defense against *S. aureus* invasion (2, 3, 17). However, pathogenic bacteria have developed defense mechanisms to evade immune recognition. Many pathogenic microbes produce CPs that mask their surface structures, where most PRR ligands are displayed (26). In this study, we examined the recognition of various phenotypes of *S. aureus* by the CTLs MGL, langerin, MBL, and SP-D and explored the influence of CP and WTA on this interaction.

Our findings indicated that MGL-expressing HEK293 cells did not bind any of the *S. aureus* or *S. pneumoniae* strains tested. We included two nonencapsulated *S. pneumoniae* strains ATCC12213 and C-mutant that produce WTA (also known as C-polysaccharide) and LTA (also known as F-antigen), which would be exposed in the absence of CP. WTA and LTA of *S. pneumoniae* have identical chains with 4 to 8 repeating units of AATGalNAc, glucose, ribitol-phosphate, and two GalNAc (45). Thus, the terminal GalNAc could be a ligand for MGL. However, we did not detect any binding by MGL to *S. pneumoniae* ATCC12213 or C-mutant. Langerin-expressing HEK293 cells, like MGL-expressing HEK293 cells, did not bind any of the *S. pneumoniae* strains. Thus, langerin, and MGL do not recognize the WTA or LTA of *S. pneumoniae*. Thus, neither of the two tested CTLs can recognize encapsulated or non-capsulated *S. pneumoniae* strains. MGL has been shown to bind to the *S. aureus* strains from the ST395 lineage, which has a different WTA structure than most *S. aureus* strains, decorated with α-GalNAc modifications conferred by the glycosyltransferase TarN (46).

Langerin-expressing HEK293 cells did bind to a collection of different *S. aureus* strains. Additionally, by utilizing the aggregating abilities of SP-D, we could show a calcium-dependent interaction with *S. aureus.* We observed that centrifugation of aggregated *S. aureus* disrupts the bacteria, rendering them unsuitable for flow cytometry experiments involving washing steps (data not shown). Only a few papers have examined the interaction of SP-D with *S. aureus*. Hartshorn et al. (47) showed that SP-D induced aggregation of an acapsular *S. aureus* strain. However, it was later shown by Bufler et al. (48) that SP-D did not aggregate any of the 10 strains tested. This could, however, be due to CP production since the strains were grown on plates rather than in broth. Furthermore, it has previously been described that purified peptidoglycan from *S. aureus* is a target of SP-D (49). Additionally, both MBL and langerin ECD were shown to interact with non-encapsulated *S. aureus*. However, we found that the presence of CPs convincingly shields *S. aureus* from recognition by all three CTLs, langerin, SP-D, and MBL. The effect of CP was more pronounced for MBL binding and SP-D aggregation than for the langerin ECD binding.

The importance of CP production for *S. aureus* virulence has been questioned, primarily due to the spread of the virulent non-encapsulated USA300 lineage in America (50). Furthermore, opposing results exist regarding the effect of encapsulation on *S. aureus* virulence. Some animal infection models show that encapsulated *S. aureus* demonstrate enhanced virulence (29, 51–53), whereas others show decreased virulence (31, 54, 55). Although the regulation of CP production has been studied extensively, it is still unclear which conditions *in vivo* promote the generation of CP. As we illustrated here, the production of CP by *S. aureus* is highly dependent on the bacterial culture conditions. CP production occurs in the post-exponential phase of *S. aureus* growth, and it is heterogeneous in an *S. aureus* population (56). Nonetheless, CP can shield *S. aureus* from opsonization and phagocytosis (26, 57) and, as we show here, can impede recognition of the microorganism by several PPRs. On the other hand, CP can block factors critical for colonization (28, 58), such as WTA, which can interact with epithelial and endothelial cells via the host proteins SREC-1 (59) and LOX-1 (60).

WTA is important for several reasons, including adhesion and colonization (61), cell wall integrity, antibiotic resistance, and immune interactions (62). Here, we confirm its role as a target for MBL. Although it has been reported that the WTA glyco-profile affects langerin binding (17, 22), our data do not entirely correlate with these findings. First, we cannot confirm the strict dependency of the β-GlcNAc orientation as compared to the α-GlcNAc orientation for both the langerin ECD and with langerin-expressing HEK293 cells, since we do not observe a difference in the interaction with the JE2*ΔtarM* and JE2*ΔtarS* mutants. Langerin binds to ligands containing a pair of vicinal equatorial hydroxyl groups in the same stereochemistry as the 3-OH and 4-OH groups of D-mannose and should in principle not be dependent on the configuration at the C1 position of the carbohydrate ring (8). On the other hand, the carbohydrate specificity of langerin may still give surprises. Naturally occurring amino acid polymorphisms of langerin can influence the glycan specificity, e.g., it may confer an increased GlcNAc preference as compared to the preference for mannose residues of the WT langerin (63). Secondly, we demonstrate langerin’s ability to interact with *S. aureus* that lack WTA, as well as with mutants that lack the glycosyltransferases TarM and TarS. Thus, our studies suggest the presence of an additional ligand on the surface of *S. aureus*. Although WTA is a highly abundant glycopolymer on the surface of *S. aureus*, a range of different surface molecules could be candidates for this additional ligand. Further research is needed to identify this second ligand, but some candidates could be the polysaccharide structure PNAG (20) or highly glycosylated surface proteins such as the serine aspartate repeat (SDR) proteins (21, 64).

Regarding the MBL recognition of *S. aureus*, we observed a distinct loss of binding of MBL to the JE2*ΔtagO* and JE2*ΔtarMS* mutants. This suggests that the WTA on *S. aureus* indeed is an authentic ligand for MBL. Additionally, MBL interacts more strongly with the JE2Δ*tarM* mutant than the WT. Similarly, the JE2Δ*tarM* mutant showed a tendency towards higher binding by langerin, as observed by Van Dalen et al. (22), presumably, since the mutant only has β-GlcNAc modifications on its WTA. Regulation of WTA biosynthesis is tightly regulated; the abundance of WTA polymers, their length, and their glycosylation profiles are all affected by environmental conditions (56). Coagulase-negative staphylococcipresent among the common skin flora produce auto-inducing cyclic peptides that inhibit the *S. aureus* global regulator *agr*, resulting in enhanced α-GlcNAc glycosylation of WTA (65). In contrast, TarS is preferentially expressed over TarM under stress-inducing conditions (66). Enhanced CTL binding seen for the JE2Δ*tarM* mutants could be due to a higher degree of β-GlcNAc glycosylation when only TarS is present.

As also covered in a recent study by Lehmann et al. (58)there is an interplay between CP and WTA during *S. aureus* infection, which is a delicate balance between immune evasion and colonization. In the host, another interplay exists between different PRRs. In this study, we have examined three different mannose-type CTLs with similar carbohydrate preferences, yet they differ in specificity. For example, SP-D has a high affinity for maltose, not seen for the other two CTLs (67). MBL and SP-D are soluble highly oligomerized proteins (**Figure 1A**); thus, multivalency is especially important for their efficient binding. Langerin, on the other hand, is found as a trimer on a cell membrane; further clustering at the membrane would increase multivalency. Furthermore, the three CTLs are found in three different compartments of the body: the skin, plasma, and lungs. *S. aureus* infections can arise in all three compartments: skin and soft tissue infections, bacteriemia, and pneumonia. The presence of several *S. aureus* binding CTLs is an example of redundancy in the innate immune system since they can all recognize *S. aureus* and even inhibit each other’s binding if co-existing. Upon recognition of a pathogen, they also have different effector functions. SP-D in the lungs will aggregate *S. aureus* and recruit phagocytes (15). *S. aureus* bound by MBL can initiate the lectin pathway of the complement system, and the bacteria will thereby be subject to opsonization (13). Recognition by langerin can lead to receptor-mediated cellular uptake and presentation to CD4^+^ T-cells (11). However, the precise downstream processes after langerin binding are not clear since langerin only has a short cytoplasmic tail and no classical signaling motifs. Our present study highlights the variability in the innate immune system against a pathogen such as *S. aureus*, with PRRs at different sites of entry and different effector functions that are vital to the battle against this pathogen.

In conclusion, we have explored the interactions among three CTLs (langerin, MBL, and SP-D) and *S. aureus*, especially focusing on the influence of the glycopolymers CP and WTA. We have shown that all three CTLs recognize *S. aureus.* However, *S. aureus* can evade recognition by CP production, shielding surface antigens like WTA. Additionally, we have confirmed that the GlcNAc decorations on WTA, both the α- and β-anomer, are ligands for MBL. However, langerin likely interacts with an additional ligand on the *S. aureus* surface. Furthermore, we have highlighted the important redundancy in the innate immune system by showing how langerin, MBL, and SP-D can compete for binding to the same bacterial structure.

## Data Availability

The raw data supporting the conclusions of this article will be made available by the authors, without undue reservation.
