# Corrigendum: Recognition of *Staphylococcus aureus* by the pattern recognition molecules langerin, mannan-binding lectin, and surfactant protein D: the influence of capsular polysaccharides and wall teichoic acid

**DOI:** 10.3389/fimmu.2025.1568032

**Published:** 2025-02-11

**Authors:** Kirstine Mejlstrup Hymøller, Stig Hill Christiansen, Anders Grønnegaard Schlosser, Uffe B. Skov Sørensen, Jean C. Lee, Steffen Thiel

**Affiliations:** ^1^ Department of Biomedicine, Aarhus University, Aarhus, Denmark; ^2^ The Centre for Cellular Signal Patterns (CellPAT), Aarhus University, Aarhus, Denmark; ^3^ Department of Inflammation Research, Department of Molecular Medicine, University of Southern Denmark, Odense, Denmark; ^4^ Division of Infectious Diseases, Department of Medicine, Brigham and Women’s Hospital and Harvard Medical School, Boston, MA, United States

**Keywords:** innate immunity, langerin, mannan-binding lectin, surfactant protein D, *S. aureus*, C-type lectins, capsular polysaccharides, wall teichoic acid

In the published article, there was an error in Affiliations 3 and 4. Instead of “^3^Division of Infectious Diseases, Department of Medicine, Brigham and Women’s Hospital and Harvard Medical School, Boston, MA, United States”, it should be “^3^Department of Inflammation Research, Department of Molecular Medicine, University of Southern Denmark, Odense, Denmark”. Instead of “^4^Department of Inflammation Research, Department of Molecular Medicine, University of Southern Denmark, Odense, Denmark”, it should be “^4^Division of Infectious Diseases, Department of Medicine, Brigham and Women’s Hospital and Harvard Medical School, Boston, MA, United States”.

The authors apologize for this error and state that this does not change the scientific conclusions of the article in any way. The original article has been updated.

