# Supplementary material for: Recognition of Staphylococcus aureus by the pattern recognition molecules langerin, mannan-binding lectin, and surfactant protein D: the influence of capsular polysaccharides and wall teichoic acid
Source: Front Immunol. 2025 Jan 7;15:1504886. doi: 10.3389/fimmu.2024.1504886 (PMC11756514; doi:10.3389/fimmu.2024.1504886)
Supplement: Supplementary file 1 [file DataSheet1.docx]

Supplementary Material

# Supplementary Figures

**
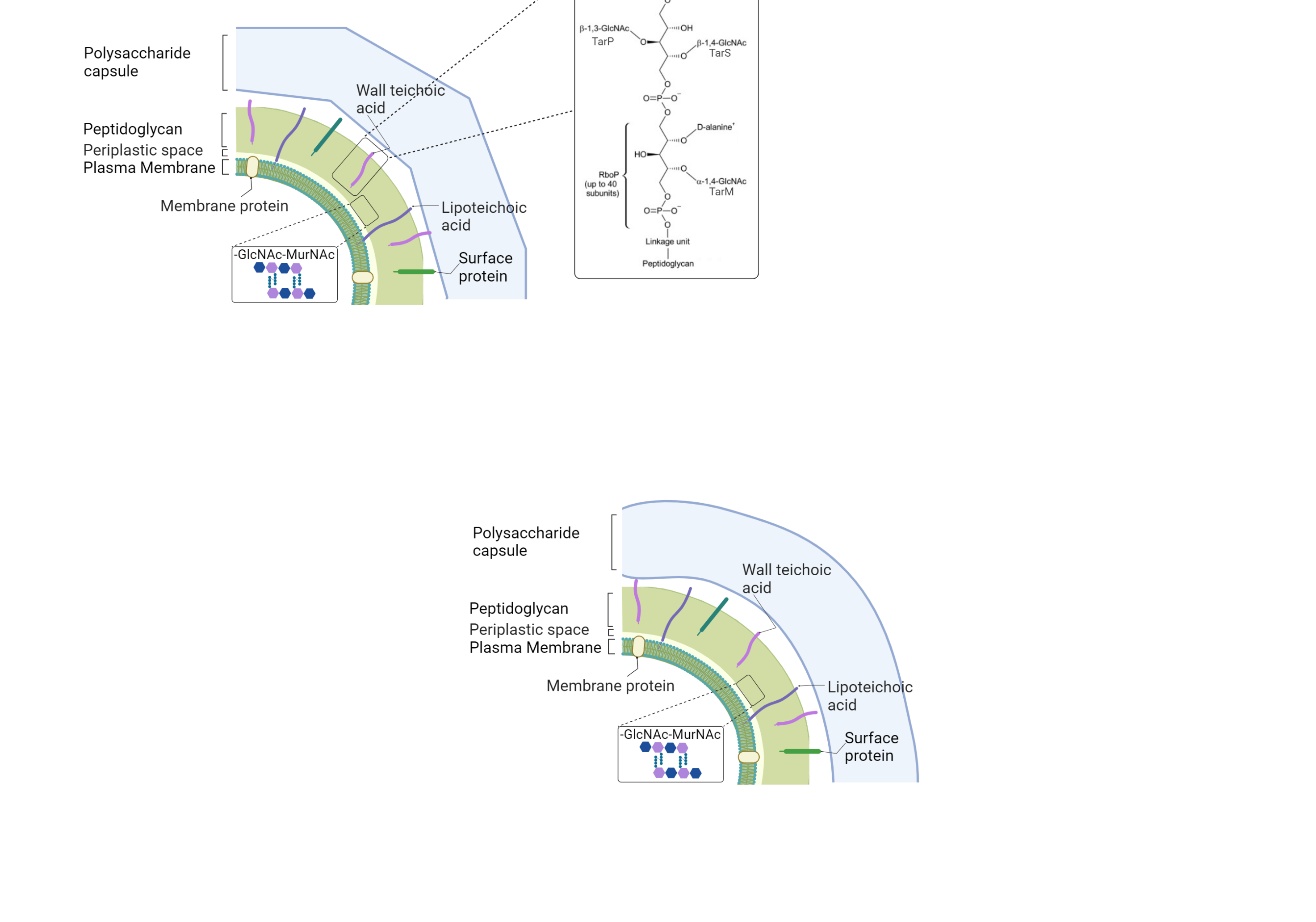
**

**Supplementary Figure S1** Schematic diagram of the cell wall of *Staphylococcus aureus.* The cell wall of the Gram-positive bacterium *S. aureus* is built up of a thick peptidoglycan layer covering the plasma membrane. The peptidoglycan consists of repeating units of N-acetylglucosamine (GlcNAc) and N-acetylmuramic acid (MurNAc) cross-linked by peptide bridges. The cell wall is also decorated with teichoic acids: either lipoteichoic acid (LTA) anchored in the plasma membrane or wall teichoic acid (WTA) covalently linked to the peptidoglycan layer. Surface proteins can be attached to the peptidoglycan layer. Most clinical *S. aureus* strains produce capsular polysaccharides that encapsulate the bacterial cells. Illustration created with Biorender.com.

**
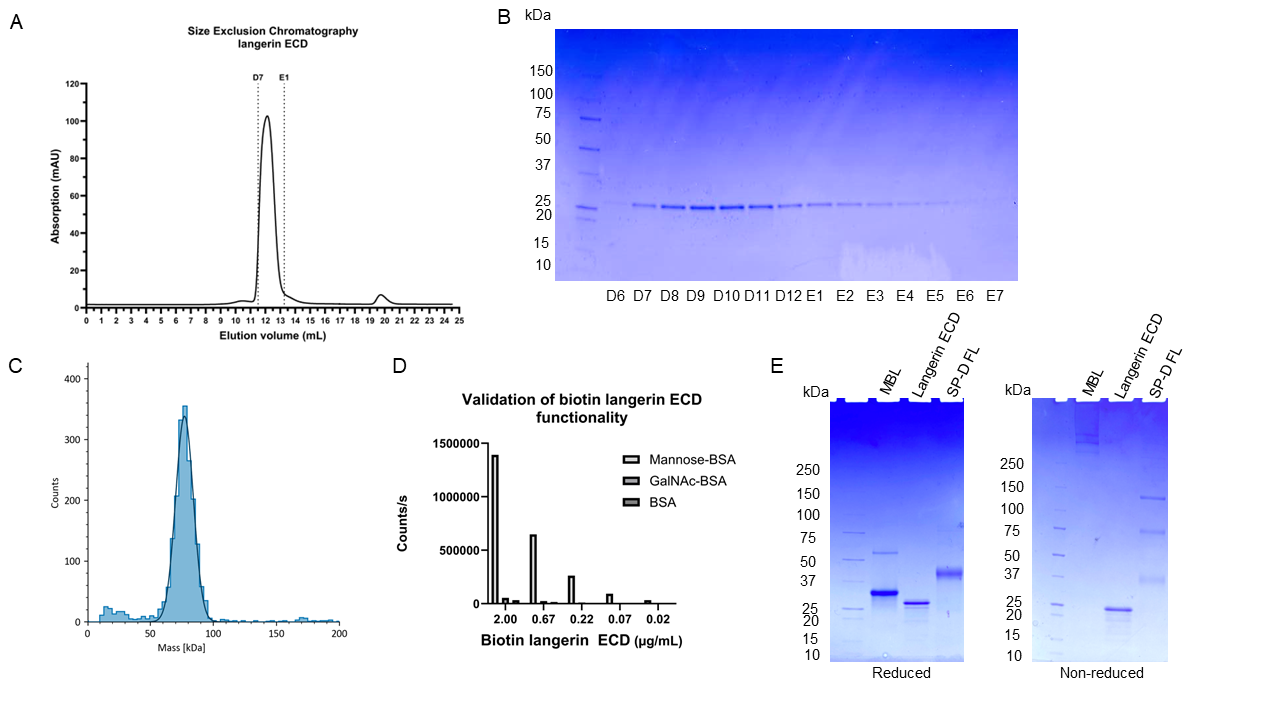
**

**Supplementary Figure S2** Purification of soluble Langerin ECD. A) Soluble langerin ECD was expressed in *E. coli*, followed by affinity chromatography on mannose-conjugated beads (see text). The eluted product was purified by size exclusion chromatography (SEC). The figure shows the elution profile on an SEC column. The y-axis gives the absorption at 280 nm (mAU), and the x-axis gives the elution volume. B) The fractions in the peak ~12 mL elution from the SEC were examined by non-reducing SDS-PAGE, and fractions D7 to E1 were pooled. The langerin monomer polypeptide chain has a theoretical molecular mass of 29 kDa. C) Mass photometry was performed to evaluate the molecular mass of soluble langerin ECD. The peak in the histogram corresponds well with the majority of soluble langerin being in a trimeric state, with a molecular weight of ~87 kDa. The x-axis gives the molecular weight of the molecules, whereas the y-axis gives the number of molecules detected. D) Functional validation of biotin langerin ECD. The binding of decreasing concentrations of biotin-langerin ECD to microtiter wells coated with mannose-BSA, GalNAc-BSA, or BSA was detected using europium labeled streptavidin (see Supplementary Methods 2.1). The Y-axis gives the amount of europium in the wells as counts per second. The data are from one individual experiment. E) SDS-PAGE of the CTLs used in this project: MBL, langerin ECD, and full-length (FL) SP-D. Samples were electrophoresed under either reduced (left) or non-reduced (right) conditions.


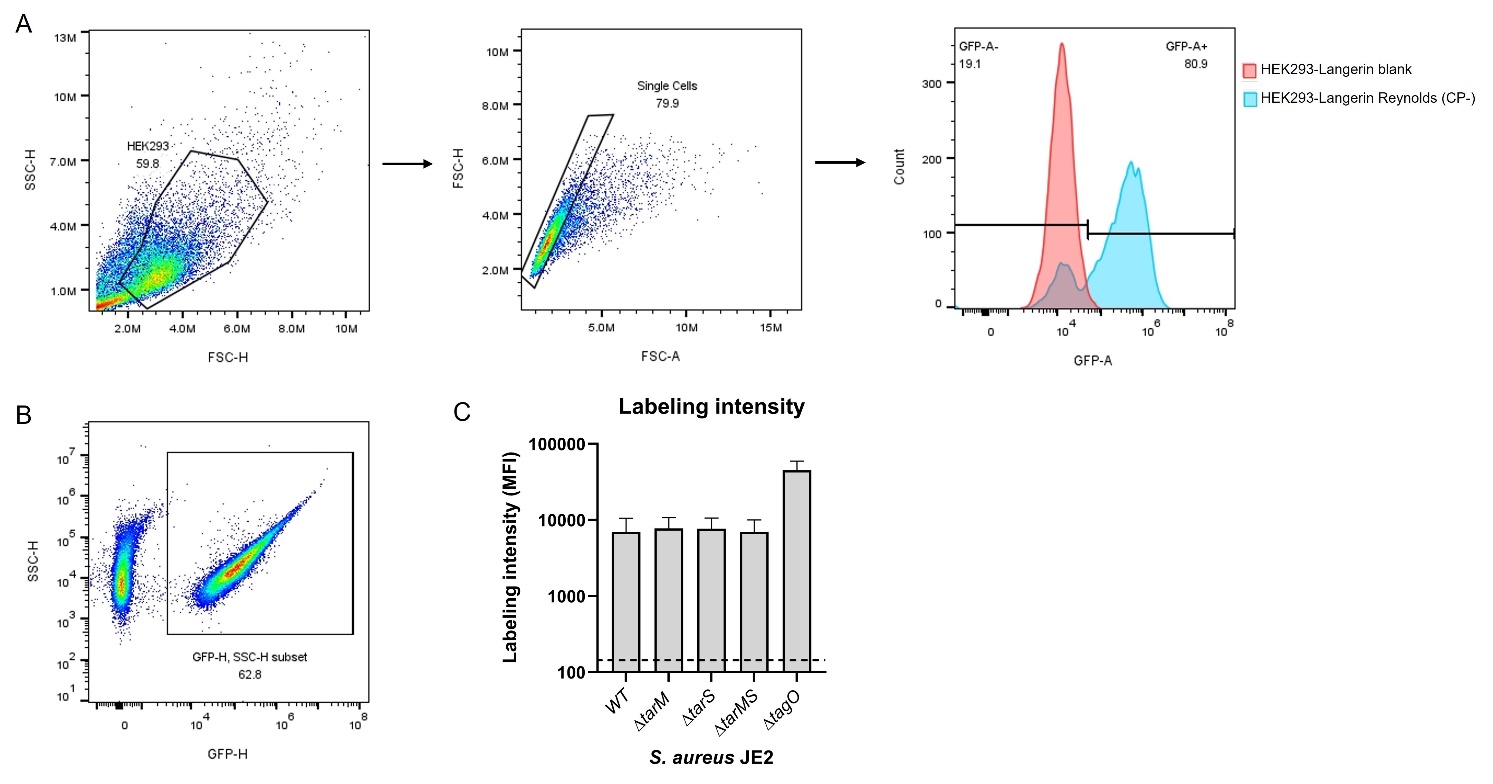


**Supplementary Figure S3** Flow cytometry gating strategy. Depicted is an example of the gating strategy for A) HEK293 cells: gating of cells by forward scatter (FSC) and side scatter (SSC) height, followed by gating of single cells by FSC area and height, and further gating on GFP/FITC positive events. B) *S. aureus* either expressing GFP or were FITC labeled are gated as the GFP or FITC height positive signals. C) The average labeling intensity of FITC labeled *S. aureus* WT JE2 or WTA mutant strains (*ΔtagO, ΔtarM, ΔtarS,* or *ΔtarMS*). Data are depicted as the mean MFI + sd of five independent FITC-labelings. The y-axis shows the MFI detected by flow cytometry (log-scale). The dotted line indicates the mean MFI of unlabeled *S. aureus* WT of five independent experiments.

***
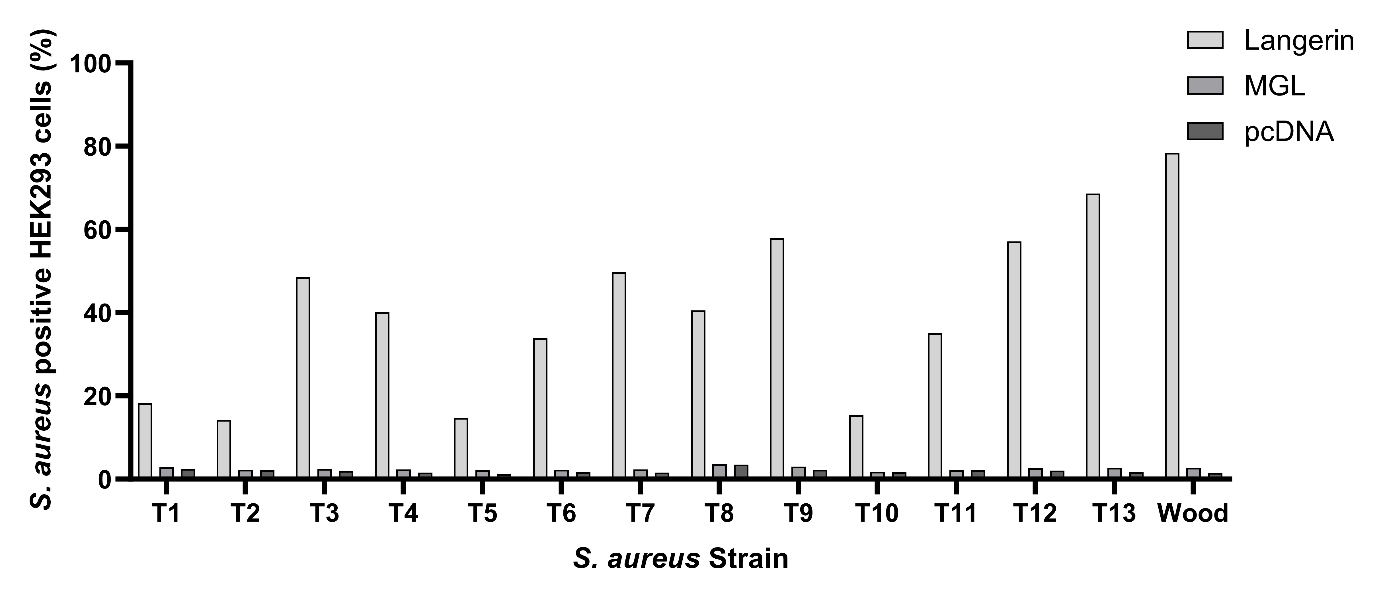
***

**Supplementary Figure S4** Binding of HEK293-Langerin and HEK293-MGL to a collection of *S. aureus* laboratory strains (see Supplementary Methods 2.2). The Wood strain is a nonencapsulated variant. A collection of different *S. aureus* laboratory strains was cultivated in Todd Hewitt broth o.n., fixed in 1% formaldehyde, and FITC-labelled before incubation with HEK293 cells expressing Langerin, MGL, or an empty vector control. *S. aureus* binding by the HEK293 cells was measured by flow cytometry. The data are depicted as the percent FITC-positive HEK293 cells in one experiment. The identity of the strains is given below the x-axis. The HEK293-Langerin cells bind all the S. aureus strains tested to varying degrees. However, HEK293-MGL cells and the empty vector control do not bind any of the *S. aureus* strains.


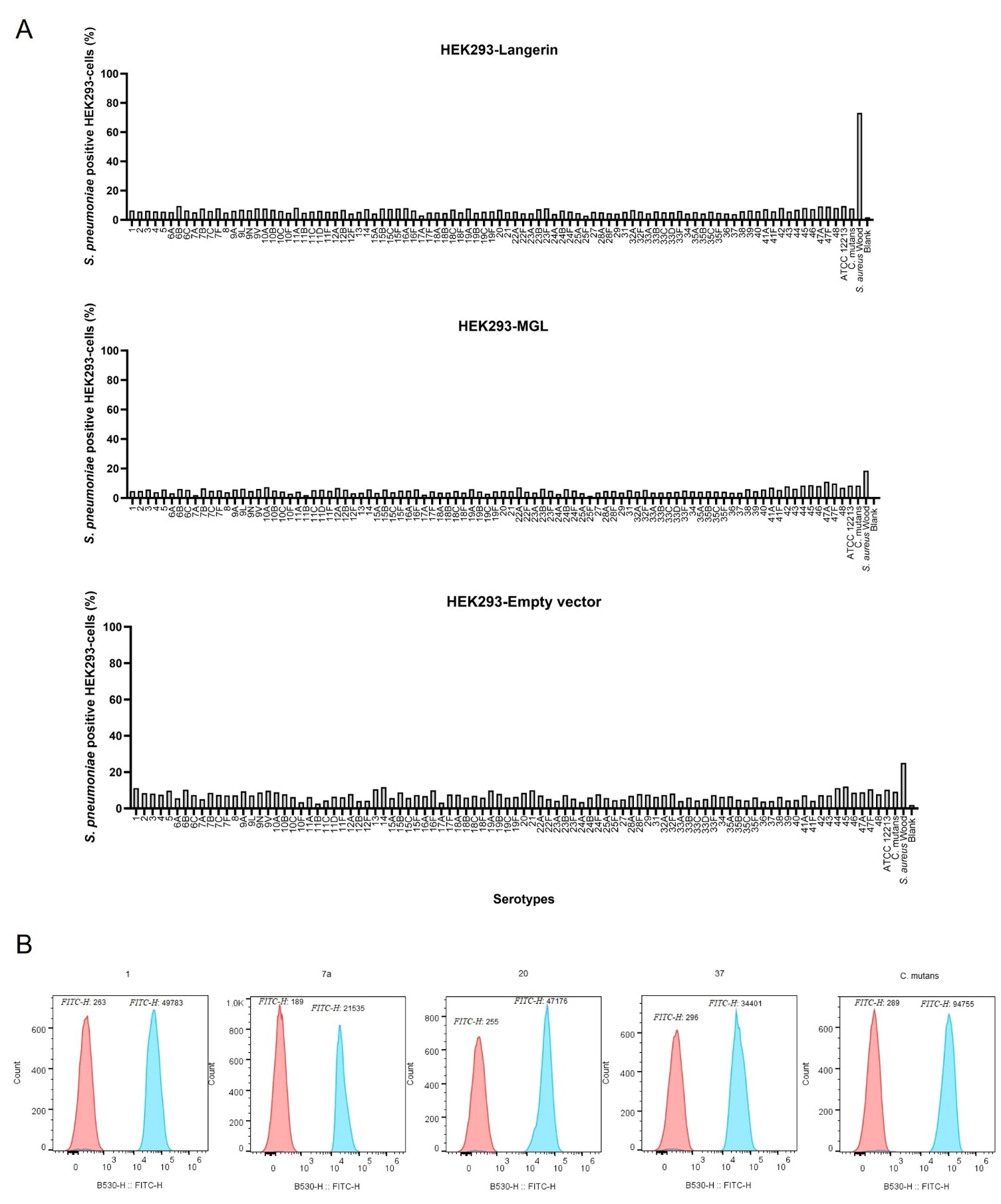


**Supplementary Figure S5** Examination of the binding of *Streptococcus pneumoniae* to HEK293-Langerin and HEK293-MGL cells (see Supplementary Methods 2.3). A) A collection of different serotypes and nonencapsulated *S. pneumoniae* strains (n=91) and one *S. aureus* strain (Wood, as positive control) were cultivated in Todd Hewitt broth for 16 hours, fixed in 1% formaldehyde, and FITC-labelled. Subsequently, the bacteria were incubated with HEK293 cells expressing Langerin, MGL, or an empty vector control. The binding of bacteria was tested by flow cytometry. Data are depicted as percent FITC-positive cells from one experiment. The identity of the strains is given below the x-axis. We find the expected binding of *S. aureus* Wood to the langerin-expressing HEK293 cells, whereas we do not see binding of either encapsulated or unencapsulated *S. pneumoniae* strains to langerin or MGL-expressing cells. B) The labeling intensity of a random sample including  *S. pneumoniae* strains: 1, 7a, 20, 37, and C. mutans was tested using flow cytometry. The red histogram represents unlabeled *S. pneumoniae* and the blue histogram represents FITC-labeled *S. pneumoniae.* The MFI is given above the histogram peak*.*


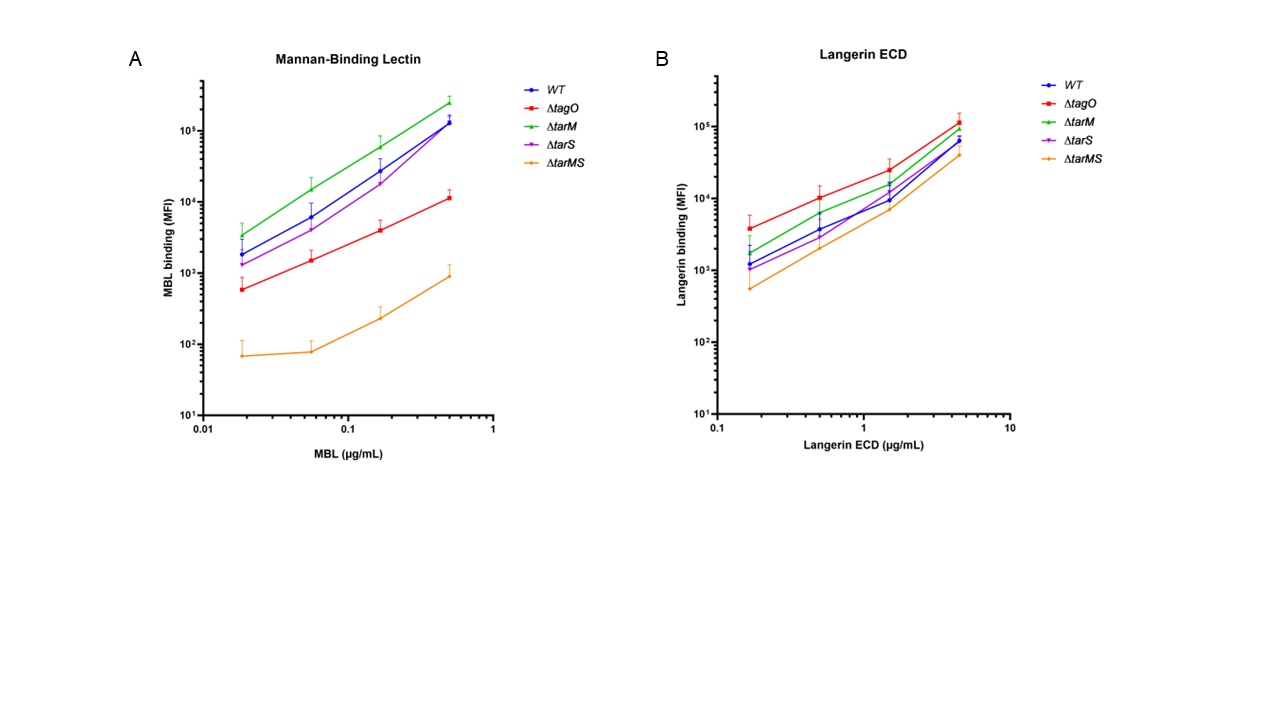


**Supplementary Figure S6** Examination of the dose-response binding of mannan-binding lectin and langerin ECD to *S. aureus* WTA mutants. The binding of increasing amounts of biotinylated mannan-binding lectin (A) or biotinylated Langerin ECD (B) to *S. aureus* WT JE2 or WTA mutant strains (*ΔtagO, ΔtarM, ΔtarS,* or *ΔtarMS)* was examined. After washing the bacteria, BV421-streptavidin was used to detect lectin binding, which was measured by flow cytometry. Data are depicted as the mean of MFI + sd of six independent experiments. The x-axis gives the concentration of langerin ECD or mannan-binding lectin (log-scale) used in the experiments, and the y-axis shows the MFI detected by flow cytometry (log-scale).

# Supplementary methods

## Molecular Mass and Functional Activity of Soluble Langerin ECD

Mass photometry was performed using a ReFeyn TwoMP Mass photometer to determine the molecular mass of the recombinant Langerin ECD. Langerin ECD was diluted to 5 nM in Tris-buffered saline (10 mM TRIS, 140 mM NaCl), and a drop was added to the sensor before recording for 60 seconds.

The functionality of soluble Langerin ECD was examined using a time-resolved immunofluorometric assay. Microtiter wells (MaxiSorp, Nunc) were coated with either mannose-BSA (NGP1108), N-acetylgalactosamine-BSA (NGP1104), or BSA (9048-46-8, Sigma-Aldrich) at 1 µg/mL in PBS. Residual binding sites in the wells were blocked with 1 mg/mL HSA in TBS for 1 h at room temperature, followed by washing with a wash buffer (TBS, 0.05% Tween-20, 5 mM CaCl_2_). A 3-fold dilution series of biotin-labeled soluble Langerin ECD was made in wash buffer starting at 2 µg/mL and added to the wells in duplicate. After incubation for 2 hours, the wells were washed, and bound soluble langerin ECD was detected using europium-labeled streptavidin (Perkin Elmer). An enhancement solution (Perkin Elmer) was added after incubation and washing. The signal from the europium, as counts per second, was measured by time-resolved fluorometry on the DELFIA-reader Victor5.

## Screening of Binding of *Staphylococcus aureus* by Lectin Expressing Cells

We tested the binding of HEK293-Langerin, HEK293-MGL, or HEK293-empty vector to 14 *S. aureus* strains named T-1, T-2, T-3, T-4, T-5, T-6, T-7, T-8, T-9, T-10, T-11, T-12, and T-13 [1] and the nonencapsulated strain Wood (NIH, Bethesda, MD, USA). The bacteria were grown in THB overnight at 37°C with agitation. The bacteria were subsequently fixed in PBS, 1% (vol/vol) formaldehyde, followed by FITC labeling. For this, the bacteria were resuspended in 100 mM NaHCO_3_ buffer pH 9. Subsequently, 10 µg/mL FITC (cat no. F1906, Invitrogen) was added, and the bacteria were incubated for 1 h at room temperature in the dark. Residual aldehyde groups were blocked with 0.1 M ethanolamine pH 9, and afterward, the bacteria were washed twice in TBS.

For the binding assay, *S. aureus* adjusted to 0.5 McFarland (McF) was incubated with Langerin- or MGL-expressing cells or empty vector HEK293 Cells (200,000 cells) in HBS+ for 30 min at room temperature. Afterward, the cells were washed twice before flow cytometry analysis was performed on a NovoCyte 3000 flow cytometer.

## Screening of Binding to *Streptococcus pneumoniae* by Lectin Expressing Cells

A range of 91 different *Streptococcus pneumoniae* strains, representing all known pneumococcal serotypes except for 11E and 6D (serotypes 1, 2, 3, 4, 5, 6A, 6B, 6C, 7F, 7A, 7B, 7C, 8, 9A, 9L, 9N, 9V, 10F, 10A, 10B, 10C, 11F, 11A, 11B, 11C, 11D, 12F, 12A, 12B, 13, 14, 15F, 15A, 15B, 15C, 16F, 16A, 17F, 17A, 18F, 18A, 18B, 18C, 19F, 19A, 19B, 19C, 20, 21, 22F, 22A, 23F, 23A, 23B, 24F, 24A, 24B, 25F, 25A, 27, 28F, 28A, 29, 31, 32F, 32A, 33F, 33A, 33B, 33C, 33D, 34, 35F, 35A, 35B, 35C, 36, 37, 38, 39, 40, 41F, 41A, 42, 43, 44, 45, 46, 47F, 47A, and 48) (the Killian collection, Department of Biomedicine, Aarhus University) [2] were screened for binding by langerin or MGL. The bacteria were cultured in THB for a maximum of 16 h at 37°C without agitation and subsequently washed and fixed in PBS, 1% (vol/vol) formaldehyde. This was followed by FITC labeling of the bacteria as described above. A random sample including the *S. pneumoniae* strains: 1, 7a, 20, 37, and C. mutans was taken out and the labeling intensity was tested using flow cytometry (Supplementary Figure S5B).

For the binding assay, 0.5 McF of each *S. pneumoniae* serotype or the *S. aureus* strain Wood (as positive control) were incubated with Langerin- or MGL-expressing HEK293 cells or empty vector HEK293 control cells (100,000 cells) in HBS+ for 30 min at 37°C. Afterward, the cells were washed twice before flow cytometry analysis on a NovoCyte Quanteon 4025 flow cytometer.

# References

1. Kjaer, T.R., et al., *Investigations on the pattern recognition molecule M-ficolin: quantitative aspects of bacterial binding and leukocyte association.* J Leukoc Biol, 2011. **90**(3): p. 425-37.

2. Kjaer, T.R., et al., *M-ficolin binds selectively to the capsular polysaccharides of Streptococcus pneumoniae serotypes 19B and 19C and of a Streptococcus mitis strain.* Infect Immun, 2013. **81**(2): p. 452-9.
